# Molecular epidemiology of *Clostridium difficile* infection in Iranian hospitals

**DOI:** 10.1186/s13756-018-0454-6

**Published:** 2019-01-14

**Authors:** Parisa Shoaei, Hasan Shojaei, Farzin Khorvash, Sayed Mohsen Hosseini, Behrooz Ataei, Hossein Tavakoli, Mohammad Jalali, J. Scott Weese

**Affiliations:** 1 Nosocomial Infection Research Center, Isfahan University of Medical Sciences, Isfahan, Iran; 2 Department of Microbiology, School of Medicine, Isfahan University of Medical Sciences, Isfahan, Iran; 3 Nosocomial Infection Research Center, Isfahan University of Medical Sciences, Isfahan, Iran; 4 Epidemiology and Biostatics Department, Isfahan University of Medical Sciences, Isfahan, Iran; 5 Infectious Diseases and Tropical Medicine Research Center, Isfahan University of Medical Sciences, Isfahan, Iran; 6 Department of Biology, University of British Columbia, Kelowna, Canada; 7 School of Food Science and Nutrition, Isfahan University of Medical Sciences, Isfahan, Iran; 8 Department of Pathobiology, Ontario Veterinary College, University of Guelph, Guelph, Canada

**Keywords:** *Clostridium difficile* infection, Molecular characterization, Risk factor, Multilocus sequence typing analysis (MLST)

## Abstract

**Background:**

*Clostridium difficile* infection (CDI) is known as one of the most important causes of nosocomial infections. The main objective of this study was to evaluate the presence of *Clostridium difficile* in the stool of hospitalized patients with diarrhea as well as in their environments.

**Methods:**

*C. difficile* isolates were characterized according to the presence of toxin genes and antibiotic resistance. Multilocus Sequence Typing Analysis (MLST) was applied for finding the genetic polymorphism and relationship among strain lineages.

**Results:**

A total of 821 samples (574 stools and 247 swabs) were collected between April 2015 and May 2017. The prevalence of *C. difficile* isolates was 28.6% (164/574) in patients and 19% (47/247) in swabs taken from medical devices, hands of healthcare workers and skin patient sites. Finally, 11.5% (66/574) toxigenic *C. difficile* strains isolated from stool samples of inpatients and 4.4% (11/247) from hands of healthcare workers and skin patient sites. All the toxigenic isolates were inhibited by a low concentration of vancomycin (MIC < 0.5 μg/ml). About 43% (33/77) and 39% of isolates were resistant to Clindamycin and moxifloxacin respectively. All isolates were susceptible to metronidazole. Toxigenic *C. difficile* strains were analyzed by MLST and were divided into 4 different STs. The detected types were ST-54 (57.9%), followed by ST-2 (31.6. %), ST-15 (5.3%) and ST-37 (5.3%), while none of the isolates were identified as ST-1 or ST-11. Significant risk factors for CDI appear to be advanced age, undergoing chemotherapy, previous surgery, and residence in the nursing home.

**Conclusions:**

CDI is common in Iran and further studies are recommended to monitor its epidemiological variations. Moreover, greater attempts must be made to encourage antibiotic stewardship by healthcare workers and the public.

## Introduction

*Clostridium difficile* is an obligate anaerobic bacillus opportunistic intestinal pathogen with the capability of large glucosylate toxins production (i.e., *tcdA*, *tcdB, cdtA,* and *cdtB*)) [1, 2]. The clinical symptoms of *C. difficile* infections (CDI) ranges from mild diarrhea to pseudomembranous colitis [3]. About 30% of CDIs are attributed to health care facilities transmission, while hospitals environmental contamination with *C.difficile* spores may account for 40% of CDI [1]. Both asymptomatic carriers and symptomatic patients can spread highly transmissible spores, leading to healthcare challenges as high-frequency recurrent CDI [1, 4, 5] The risk of CDI acquisition may increase in a larger hospital setting due to increased environmental contamination and improper disinfection [6]. There are few reports on *C. difficile* isolation from medical devices, hands of healthcare workers and its transmission to patients in the hospital settings [7]. The epidemiology of CDI has changed during the last two decades. However, both the incidence and severity of CDI have been increased in hospitals worldwide [3, 8]. Epidemics of CDI have happened in North America and Europe recently and the epidemiology of CDI in these regions is known. Circulating strains in Asia, as in other regions, have the potential to extend internationally [9]. These epidemics have increased the need for surveillance of *C. difficile* strains globally [1]. In recent epidemiologic studies, genotyping of strains play an important role in the identification of epidemic, hypervirulent genotypes and the relations between them [10]. In Iran, most of the studies have been limited to PCR based ribotyping [11, 12]. The appropriate use of this method as a genotyping tool for small-scale analyses has been confirmed, However, the main advantage of Multilocus Sequence Typing Analysis (MLST) is the unambiguous ability to compare the results obtained in the different regions via the internal database (accessible at https://pubmlst.org/cdifficile) [13–15]. The information included in the MLST database principally consists of strains from European countries, with few strains from Asia, especially from China [10, 16]. Moreover, no molecular epidemiologic study on *C. difficile* isolates from patients and their environment have been conducted in Iran using the MLST method.

The main objective of the current study was to determine the incidence of *C. difficile* in hospitalized patients and hospital environments. Additionally, we intended to study the genetic polymorphism and relationship among strain lineages using MLST as an unambiguous molecular procedure.

## Methods

### Study design

A cross-sectional study was conducted in medical wards of the university hospitals in Isfahan, the central of Iran from April 2015 and May 2017. Our study included the patients aged at least 18 years who were admitted to medical wards for different problems and acquired diarrhea in hospital. Diarrhea was determined as the passage of more than two loose or watery stools per day for at least two days [17]. A case of CDI was determined as a diarrheal patient with positive *C. difficile* culture and toxin tests [18]. Patients admitted for less than 3 days or who were diagnosed with CDI within the previous 3 months, colectomy and diarrhea on admission were excluded.

To avoid overrepresentation, only the first stool specimen from each patient was included. A total of 247 samples were collected from the dominant hand of healthcare workers, the different skin sites of patients (hand, forearm, and abdomen) and medical devices using sterile swab moistened with normal saline rotated all 5× 20 cm of the surfaces. All surfaces frequently touched by health care workers were documented. Fecal and environmental samples from patients were collected in sterile containers and were then immediately transferred into the laboratory of Infectious Diseases and Tropical Medicine Research Center, Isfahan, Iran and preserved at − 70 °C. Variables were included the demographic characteristics, type of comorbidities and antibiotic treatment in 8 weeks prior to CDI diagnosis.

#### Ethical considerations

The study was approved by the human research ethics committee of Isfahan University of Medical Sciences (the grant No. 293347), and the study was carried out in full accordance with the approved guidelines.

### Clostridium difficile culture

For culture analysis, Lemee L method was used [14]. About 2 g of stools (or samples taken by swabs) were inoculated into 10 ml of *C. difficile* moxalactam norfloxacin (CDMN) broth culture. Then the cultures were incubated anaerobically for 48 h at 37 C° using an Anoxomat system (MART Microbiology B.V., The Netherlands). Alcohol shock treatment was performed to inhibit non-sporulating organisms and enhance the isolation of *C. difficile* (a 1:1 suspension cultured sample with 95% ethanol was slowly vortexed and held at room temperature for 30 min). The pellets were inoculated by a swab into the *C. difficile* moxalactam norfloxacin agar (CDMN) supplemented with 7% sheep blood and incubated anaerobically for 48 h at 37C°. Negative cultures remained in the incubator for up to 7 days. Irregular yellowish colonies with horse-manure odour, ultraviolet fluorescence (365 nm), Gram stain morphology, malachite green for spore and biochemical reactions such as L-proline aminopeptidase test (Prodisk, Remeb, Lenexa, KS, USA) were identified as *C. difficile* strains and stored at 4 °C. These colonies were subjected to further molecular identification [12, 19].

### Molecular identification of *C. difficile*

All isolates were screened for the presence of the genes encoding toxin A and B (*tcd A* and *tcd B*), binary toxin (*cdt A, cdt B*) and triose phosphate isomerase (*tpi*) as described by Stubbs et al. and Lemee et al. [14, 20]. DNA extraction was performed using the procedure performed in the study by Pitcher et al. (1989). Cultures of *C. difficile* strains grown in BHI broth were centrifuged and cells were treated with lysozyme and resuspended in TE (Tris, 10 mM; EDTA, 50 mM; pH 8.0). Then guanidine isothiocyanate and ammonium acetate were added to the mixture and Chloroform/isoamyl alcohol (24:1). The DNA was precipitated and washed with ethanol [21].

Multiplex PCR amplification performed in a thermocycler (Eppendorf, Germany). An amount of 25 μl of reaction mixture contained 1× PCR buffer, 250 μM of each dNTPs, 10 pM of primers (*tcdA, tcd B*), 5 pM of primers (tpi), 1 U Taq polymerase (Cina Gene, Iran) and 100 ng of DNA. Amplification was carried out in a touchdown protocol [12, 20]. *Clostridium difficile* ribotype 027 was used as a positive control for molecular and microbiological analysis and *C. perfringens* 450 MTCC (Microbial Type Culture Collection) was used as the negative control [20].

### Multilocus sequence typing (MLST)

MLST was carried out and analyzed for *C. difficile* strains according to the previous studies. MLST with seven housekeeping genes *(adk, atpA, dxr, glyA, recA, sodA,* and *tpi*) was performed on detected isolates as described previously by Griffiths et al. [13]. Sequence types (STs) were analyzed by constructing a dendrogram based on the UPGMA (Unweighted Pair Group Method with Arithmetic mean) clustering algorithm available in the BioNumerics software [22].

### Antibiotic susceptibility testing

Antimicrobial susceptibility of toxigenic *C.difficile* isolates was determined using the Etest strips (bioMérieux, France) for vancomycin, metronidazole, moxifloxacin, fusidic acid, rifampin, and clindamycin. Briefly, the Etest was performed by inoculating the surface of pre-reduced Brucella Agar plates containing vitamin K1 (0.5 mg/mL), haemin (5 mg/mL) and 5% defibrinated sheep red blood cells with 1 McFarland standard matched *C. difficile* inoculum. The plates were incubated at 37 °C for 48 h under anaerobic conditions. The breakpoints and interpreting of the minimum inhibitory concentrations (MICs) were carried out according to the Clinical Laboratory Standards Institute (CLSI 2012) and the European Committee on Antimicrobial Susceptibility Testing (EUCAST) guidelines (http://www.clinical_breakpoints/clinical_breakpoints/clostridiumdifficle/ *difficile*). *Clostridium perfringens* MTCC 13124 and Streptococcus sp. MTCC 689 strains were included in each run as controls. The breakpoints used were 8 μg/ ml for Clindamycin, 4 μg/ ml for moxifloxacin; 2 μg/ ml, for vancomycin and 32 μg/ ml for metronidazole; rifampin; fusidic acid as described previously [23, 24]. The antimicrobial agents tested were chosen because of the emergence of reduced susceptibility.

### Statistical analysis

Data were expressed as count and percentage. All probabilities were two-tailed and a *P*-value of < 0.05 was defined as statistically significant. The logistic regression model was used to determine clinical factors associated with *C.difficle* infection. First, a univariate logistic regression model was fitted on each clinical factors, and then a multivariate regression model with adjustment for the effects of other covariates was used. Variables that were significant in univariate models (*P* < 0.05) were entered into the multivariate model. Selection of variables in the multivariate model was based on a stepwise procedure. Statistical analysis was performed using the statistical software (SPSS, version 16).

## Results

**Fig. 1:**
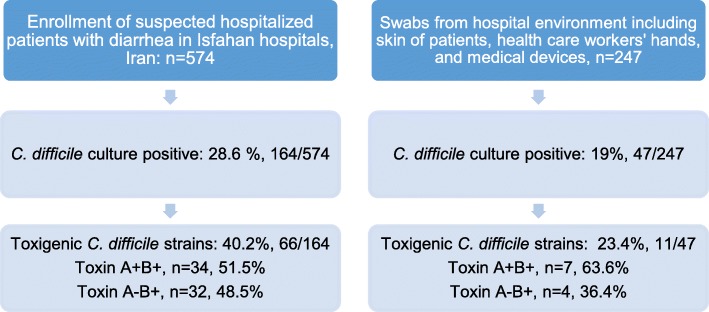
Flowdiagram illustrating the number of patients and swab samples included in the study

Among the 574 enrolled patients, 164 (28.6%) were *C. difficile* culture positive and the *tpi* gene was recovered from these stool specimens. Based on PCR amplification of *tcdA* and *tcdB*, 66 isolates (40.2%) carried one or both of these genes and were considered toxigenic, while the remaining 98 isolates (59.8%) were nontoxigenic. (Fig. 1). There were no deletions found in *tcdC* genes from all toxigenic isolates examined. No isolate tested positive for the binary toxin genes *cdtA* and *cdtB*.

Mean age of CDI cases was 55.4 years (standard deviation 19.4 years) and mean age of Non-CDI cases was 44 years(standard deviation 15.5 years). Risk of CDI increased by nearly 4% per year (OR 1.03; 95% CI, 1.01–1.05; *P* < 0.001). The use of antibiotic was identified in all patients in the 8 weeks prior to CDI diagnosis. Patients were treated with one to four antibiotics, about 96% and 63/5% of them had been used more than one and two antibiotics, respectively (Table 1).

**Table 1:** Clinical characterization of 574 hospitalized patients with nosocomial diarrhea admitted to the university hospitals, Isfahan, Iran

Characteristics	CDI patients *n* = 66	Non CDI Patients *n* = 508	
	Toxigenic *C.difficile* Isolates, (A+B+, A-B+),	Negative *C.difficile* strains *n* = 410	Non-toxigenic *C.difficile* strains (A-B-), *n* = 98
	Count (%)	Count (%)	Count (%)
Sex			
Male	31 (47.0)	208 (53.2)	49(52.1)
Antibiotic treatment within 8 weeks			
Aminoglycosides	20 (30.3)	70 (17.6)	20 (21.1)
Vancomycin	0	3 (0.7)	0
Cephalosporin	24 (36.4)	178 (44.8)	42 (44.2)
Metronidazole	11 16.7)	6 (1.5)	2 (2.1)
Clindamycin	31 (46.9)	137 (34.5)	31 (32.6)
Gastroenteritis diseases	35 (53.0)	44 (11.1)	14 (14.9)
Residence in nursing home	14 (21.2)	7 (1.8)	3 (3.2)
Chronic renal insufficiency	26 (39.4)	47 (11.9)	16 (17.0)
Previous Surgery	50 (75.8)	137 (34.7)	51 (53.7)
Chemotherapy	16 (24.2)	25(6.0)	13 (13.3)
Ward			
Internal^a^	28 (42.4)	293 (73.4)	61(64.2)
ICU	38 (57.6)	106 (26.6)	34 (35.8)

^a^Internal ward(gasteroenterology, Infectious diseasese, Diabetes,…)

The stepwise multivariate logistic regression model revealed that the following parameters were found statistically significant between cases of CDI and cases without CDI: Advanced age, previous surgery, chemotherapy or residence in a nursing home (Table 2). Among the 274 screened samples taken from hospital environment, 47(19%) were positive for *C. difficile* colonization and 4.4% (11/247) toxigenic strains. Toxigenic *C.difficile* strains were detected from different skin sites (hands and abdomens) of 7 CDI patients and 4 of healthcare workers hands (Table 3).

**Table 2:** Variables significantly associated with *C.difficile* infection among 574 patients with nosocomial diarrhea

Risk factors	OR (95% CI)	*p*-value
Age	0.97 (0.95–1.0)	0.004
Residence in nursing home	3.96 (1.0–15.6)	0.049
Chronic renal insufficiency	2.69 (1.3–5.6)	0.008
Previous surgery	4.23 (2.1–8.6)	< 0.001
Chemotherapy	2.96 (1.3–6.7)	0.01

**Table 3:** Summary of positive surface *Clostridium difficile* cultures

Category	Samples (*n* = 247)	Non-toxigenic *C.difficile* colonization *n* (%)	Toxigenic CdC^a^, *n* (%)
HCW’^b^ hands	73	21 (28.8)	4 (5.4)
Skin of patients	82	17 (20.7)	7 (8.5)
ICU devices:	52	4 (7.7)	0
Bed sheets	40	5 (12.5)	0

^*a*^*Clostridium difficile* colonization (CdC),

^b^health care worker (HCW)

### Molecular epidemiology of the isolates

**Fig. 2:**
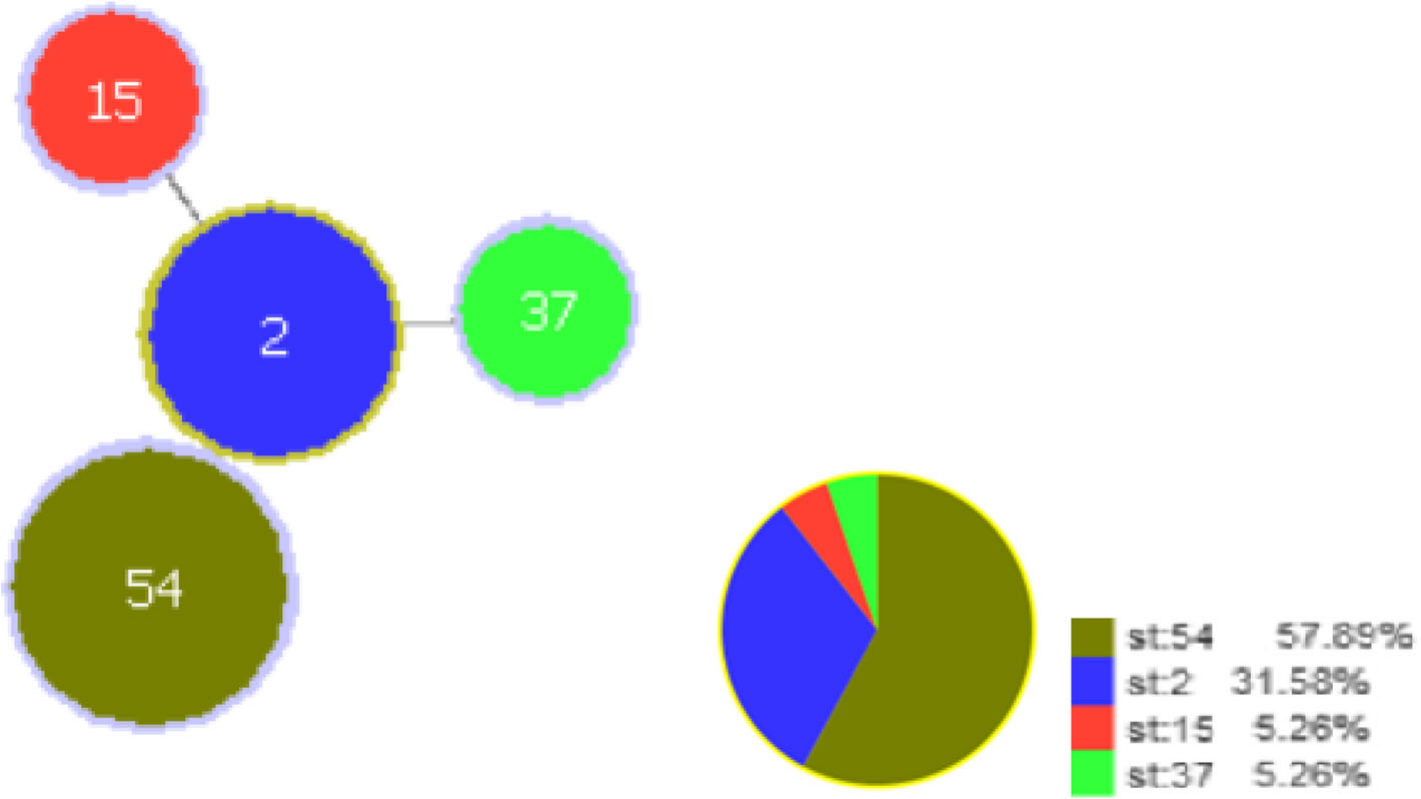
Distribution of *C. difficile* sequence types(ST) identified by MLST

An additional two isolates were obtained from collection reference laboratories of *C. difficile* analysis (held at Canada, University of Guelph). They represented two different PCR ribotypes (027, 078) selected to validate the MLST scheme analyses. Toxigenic *C. difficile* strains from different hospitals were analyzed by MLST and divided into 4 different STs (Fig. 2).

The detected types were ST-54 (57.9%), followed by ST-2 (31.6%), ST-15 (5.3%), and ST-37 (5.3%), but none of the isolates was identified as ST-1 (BI/NAP1/027) or ST-11(ribotype 078). We found a correlation between the toxin genotypes and STs. All of the ST-54 strains were toxin type A^+^B^+^, and all of the ST-2 strains were A^−^B^+^. Some relations were observed between the toxin profile and the wards. For instance, ST-54 and ST- 2 were the major types in the ICU and together accounted for 60% of the strains. Among other wards especially in gastroenterology ward, ST-54 and ST-2 were the most frequent STs and both equally represented at 35.5% of the strains. There was no significant difference between males and females in the prevalence of ST types (*P* > 0.05).

### Antibiotic susceptibility of *C. difficile*

We used E test strips to determine the minimum inhibitory concentration (MIC) of each antibiotic in the toxigenic isolates for six antibiotics. All the isolates were inhibited by a low concentration of vancomycin (MIC < 0.5 μg/ml). Two isolates, from 2 ICU patients aged 70 and 72 years, were resistant to fusidic acid, while the remaining isolates (97.4%) proved susceptible to it. About 42.8% (33/77) of isolates were resistant to Clindamycin, while it was about 38.9% for Moxifloxacin (30/77). Only 11.7% of isolates (9/77) were resistant to rifampin. All isolates were susceptible to metronidazole. There were no inner colonies suggestive of heteroresistance within the zones of inhibition around the metronidazole E- test strips (Table 4). There was no correlation between the toxin type and antibiotic resistance (*P* > 0.05).

**Table 4:** MIC for antimicrobial agents tested against 77 *C. difficile* isolates

Antibiotic	MIC (μg/ml)	Resistant	Break points (μg/ml)
	Range	no. (%)	
Clindamycin	0.25 > 256	33 (42.8)	≥ 8
Metronidazole	0.125–8	0	≥ 32
Rifampin	0.002 > 32	9 (11.7)	≥ 32
Moxifloxacin	0.5 > 32	30 (38.9)	≥ 4
Vancomycin	0.016–0.5	0	> 2
Fusidic acid	0.016 > 8	2 (2.6)	≥ 32

## Discussion

Currently, *C. difficile* infection (CDI) is regarded as a widespread issue worldwide because of its increasing morbidity and mortality. The range of this infection is variable from mild diarrhea to pseudomembranous colitis especially among the elderly population who have been exposed to antibiotics [10]. CDI epidemics have indicated the need for surveillance of the international prevalence of *C. difficile* strains. There are limited epidemiological studies on *C. difficile* in Iran. The prevalence of CDI in our study was lower than those reported in Western countries and China [25, 26]. The prevalence of CDI at hospitals in Kuwait was reported 9.7% in 2003, 7.8% in 2004 and 7.2% in 2005 with 002, 001, 126 and 140 RTs were the most frequent ones found [11]. In a similar study performed in Qatar, CDI prevalence was reported around 8% and RT 258 was shown to be the predominant ribotype [27].In European Countries, Binary toxin (CDT) has been found in 4–12% of toxigenic *C. difficile* samples associated with higher mortality and recurrence rate of CDI [28]. We found a high prevalence of non-toxigenic strains, the absence of binary toxin producers and hypervirulent RTs 027 and 078 in the current study. Our findings were in accordance with similar studies in Asia showing a low prevalence of binary toxin among isolates in various hospitals in China, Thailand, Korea and Japan [10]. This may be the result of different circulating *C. difficile* strains in Asia.

Our phylogenetic analysis showed that ST-54 and ST-2 were the most prevalent strains. Therefore we proposed a low genetic diversity of toxigenic strains in Isfahan, Iran. Other similar studies in Europe, the Middle East, and North America previously identified ST2 as a common clinical strain all around the world [29]. Studies performed in China and France showed various STs in A^−^B^+^ strains, yet the most prevalent STs were ST-2, ST-54, ST-37, and ST-35 in China and ST-1 in France. This might be due to different geographical locations where the study was carried out [9, 10, 26].

Most of CDI in the current study were found to be due to A^+^B^+^ strains (ST-54). Recent studies have reported an increasing number of infections due to A-B+ strains especially ST-37 in Asia though such strains do not produce a binary toxin [10]. Due to unavailability of *C. difficile* culture and toxin testing in many hospitals in Iran, awareness of circulating strains and their prevalence is not high enough.

We could not establish a correlation between the hospitals and detected genotypes. Our isolates were genotyped into 4 STs, which were not specific to Iran, a finding that suggests the worldwide spread of some lineages. All strains of ST54 isolates were toxin type A + B+ and all strains ST2 were toxin type A-B+. In addition, there was no correlation between the genotype and CDI risk factors in our study. Based on our multivariate regression model results, patients with CDI were significantly older and had more comorbidities than the non-CDI group. Several previous studies have noted the association between recent hospitalization, advanced age, severe diseases and a higher risk of acquiring CDI [9, 26, 30, 31]. All identified toxigenic stains in this study showed susceptibility to vancomycin and metronidazole. Other similar studies showed that toxigenic *C.difficile* strains commonly revealed high susceptibility to these most common choices for CDI treatment [28]. In two studies conducted in China, the rate of resistance to moxifloxacin was reported at 46.4 and 13.1% [26]. The strong correlation between CDI incidence and resistance to these antibiotics showed that it is necessary to restrict the use of fluoroquinolones to reduce CDI [31]. We have found that *C. difficile* frequently contaminated hands of HCWs after caring for patients with CDI (*p* < 0.01). Other studies showed that skin contamination persisted in many patients after resolution of diarrhea and was easily acquired on investigators hands [32] The same STs (STs 54, 2, 15) were detected from the health care workers hands, the skin of patients and their stool samples. One similar study already conducted in Iran also showed that the occurrence of *C. difficile* isolates with the same RTs in gastrointestinal imaging devices and stool specimens [11]. While a contaminated environment is a significant contributor to infection, skilled healthcare workers and effective infection control measures in the patient hospital rooms can reduce the burden of CDI in health care facilities.

There were a number of limitations to our study including difficulties in the sequencing of all *C. difficile* isolates, which led to undermining the true prevalence and diversity of *C. difficile* STs. Due to the quick discharge of many of our patients; we were not able to analyze treatment and outcome characteristics of all patients with CDI.

Since the incidence of CDI was relatively high at the provincial level, a countrywide CDI surveillance (with a long period and large population size) is warranted to analyze other risk factors of CDI and contamination.

## Conclusion

According to the results, a relatively high infection rate of toxigenic and non-toxigenic strains of *clostridium difficile* was observed in our patients and found to exist in their environment. More studies are recommended to monitor its epidemiological change and greater attempts must be made to encourage antibiotic stewardship by healthcare professionals and the public.

## Abbreviations

CDI: *Clostridium difficile* infection
CDMN: *C. difficile* moxalactam norfloxacin
MIC: Minimum inhibitory concentration
MLST: Multilocus sequence typing analysis
RT: Ribotype
ST: Sequence type

## References

[1] Gil F, Lagos-Moraga S, Calderon-Romero P, Pizarro-Guajardo M, Paredes-Sabja D (2017). Updates on *Clostridium difficile* spore biology. Anaerobe.

[2] Lawson PA, Citron DM, Tyrrell KL, Finegold SM (2016). Reclassification of *Clostridium difficile* as *Clostridioides difficile* (hall and O’Toole 1935) Prévot 1938. Anaerobe.

[3] Walker AS, Eyre DW, Wyllie DH, Dingle KE, Harding RM, O’Connor L (2012). Characterisation of *Clostridium difficile* hospital ward–based transmission using extensive epidemiological data and molecular typing. PLoS Med.

[4] Kumar N, Miyajima F, He M, Roberts P, Swale A, Ellison L (2016). Genome-based infection tracking reveals dynamics of *Clostridium difficile* transmission and disease recurrence. Clin Infect Dis.

[5] Putsathit P, Maneerattanaporn M, Piewngam P, Kiratisin P, Riley TV (2017). Prevalence and molecular epidemiology of *Clostridium difficile* infection in Thailand. New Microbes New Infect.

[6] Jou J, Ebrahim J, Shofer FS, Hamilton KW, Stern J, JHJic H (2015). Environmental transmission of *Clostridium difficile*: association between hospital room size and *C. difficile* Infection. Infect Control Hosp Epidemiol.

[7] Gerding DN, Muto CA, Owens RC, Jr. Measures to control and prevent Clostridium difficile infection. Clin Infect Dis 2008;46 Suppl 1(Supplement_1): S43–S49. 10.1086/521861 18177221

[8] Ofosu A (2016). *Clostridium difficile* infection: a review of current and emerging therapies. Ann Gastroenterol.

[9] Collins DA, Hawkey PM, Riley TV (2013). Epidemiology of *Clostridium difficile* infection in Asia. Antimicrob Resist Infect Control.

[10] Chen YB, Gu SL, Wei ZQ, Shen P, Kong HS, Yang Q (2014). Molecular epidemiology of *Clostridium difficile* in a tertiary hospital of China. J Med Microbiol.

[11] Azimirad M, Krutova M, Nyc O, Hasani Z, Afrisham L, Alebouyeh M (2017). Molecular typing of *Clostridium difficile* isolates cultured from patient stool samples and gastroenterological medical devices in a single Iranian hospital. Anaerobe.

[12] Jalali M, Khorvash F, Warriner K, Weese JS (2012). *Clostridium difficile* infection in an Iranian hospital. BMC Res Notes.

[13] Griffiths D, Fawley W, Kachrimanidou M, Bowden R, Crook DW, Fung R (2010). Multilocus sequence typing of *Clostridium difficile*. J Clin Microbiol.

[14] Lemee L, Dhalluin A, Pestel-Caron M, Lemeland J-F, Pons J-L (2004). Multilocus sequence typing analysis of human and animal *Clostridium difficile* isolates of various toxigenic types. J Clin Microbiol.

[15] Urwin R, Maiden MC (2003). Multi-locus sequence typing: a tool for global epidemiology. Trends Microbiol.

[16] Yan Q, Zhang J, Chen C, Zhou H, Du P, Cui Z (2013). Multilocus sequence typing (MLST) analysis of 104 C*lostridium difficile* strains isolated from China. Epidemiol Infect.

[17] Goudarzi M, Goudarzi H, Alebouyeh M, Azimi Rad M, Shayegan Mehr FS, Zali MR (2013). Antimicrobial susceptibility of *clostridium difficile* clinical isolates in Iran. Iran Red Crescent Med J.

[18] Garey KW, Dao-Tran TK, Jiang ZD, Price MP, Gentry LO, Dupont HL (2008). A clinical risk index for *Clostridium difficile* infection in hospitalised patients receiving broad-spectrum antibiotics. J Hosp Infect.

[19] Rodriguez C, Taminiau B, Avesani V, Van Broeck J, Delmee M, Daube G (2014). Multilocus sequence typing analysis and antibiotic resistance of *Clostridium difficile* strains isolated from retail meat and humans in Belgium. Food Microbiol.

[20] Stubbs S, Rupnik M, Gibert M, Brazier J, Duerden B, Popoff M (2000). Production of actin-specific ADP-ribosyltransferase (binary toxin) by strains of *Clostridium difficile*. FEMS Microbiol Lett.

[21] Pitcher D, Saunders N, Owen R (1989). Rapid extraction of bacterial genomic DNA with guanidium thiocyanate. Lett Appl Microbiol.

[22] Zaiss NH, Rupnik M, Kuijper EJ, Harmanus C, Michielsen D, Janssens K (2009). Typing *Clostridium difficile* strains based on tandem repeat sequences. BMC Microbiol.

[23] Tickler IA, Goering RV, Whitmore JD, Lynn AN, Persing DH, Tenover FC (2014). Strain types and antimicrobial resistance patterns of *Clostridium difficile* isolates from the United States, 2011 to 2013. Antimicrob Agents Chemother.

[24] Wayne P. Clinical and laboratory standards institute. Performance standards for antimicrobial susceptibility testing 2011. 31339681

[25] Lessa FC, Mu Y, Bamberg WM, Beldavs ZG, Dumyati GK, Dunn JR, et al. Burden of *Clostridium difficile* infection in the United States. N Engl J Med. 2015;372(9):825–34. 10.1056/NEJMoa1408913 PMC10966662 25714160

[26] Yan J, Liang J, Lv T, Gu S, Jiang T, Huang L, et al. Epidemiology of *Clostridium difficile* in a county level Hospital in China. Jundishapur J Microbiol. 2017;10(6).

[27] Al-Thani AA, Hamdi WS, Al-Ansari NA, Doiphode SH, Wilson GJ (2014). Polymerase chain reaction ribotyping of *Clostridium difficile* isolates in Qatar: a hospital-based study. BMC Infect Dis.

[28] Ngamskulrungroj P, Sanmee S, Pusathit P, Piewngam P, Elliott B, Riley TV (2015). Molecular epidemiology of *Clostridium difficile* infection in a large teaching hospital in Thailand. PLoS One.

[29] Michael K, No D, Dankoff J, Lee K, Lara-Crawford E, Roberts MC (2016). *Clostridium difficile* environmental contamination within a clinical laundry facility in the USA. FEMS Microbiol Lett.

[30] Jin D, Luo Y, Huang C, Cai J, Ye J, Zheng Y, et al. Molecular epidemiology of *Clostridium difficile* infection in hospitalized patients in eastern China. J Clin Microbiol. 2017;55(3):801–10. 10.1128/JCM.01898-16 PMC5328448 27974547

[31] Kim J, Kang JO, Kim H, Seo MR, Choi TY, Pai H (2013). Epidemiology of *Clostridium difficile* infections in a tertiary-care hospital in Korea. Clin Microbiol Infect.

[32] Bobulsky GS, Al-Nassir WN, Riggs MM, Sethi AK, CJJCid D. *Clostridium difficile* skin contamination in patients with C difficile–associated disease. Clin Infect Dis. 2008;46(3):447–50. 10.1086/525267 18181742

